# Mapping of a Quantitative Trait Locus for Stay-Green Trait in Common Wheat

**DOI:** 10.3390/plants14050727

**Published:** 2025-02-27

**Authors:** Xin Li, Xin Bai, Lijuan Wu, Congya Wang, Xinghui Liu, Qiqi Li, Xiaojun Zhang, Fang Chen, Chengda Lu, Wei Gao, Tianling Cheng

**Affiliations:** Shanxi Key Laboratory of Crop Genetics and Molecular Improvement, College of Agronomy, Shanxi Agricultural University, Taiyuan 030031, China; lixin@sxau.edu.cn (X.L.);

**Keywords:** wheat, stay green, flag leaf, chlorophyll content, QTL mapping

## Abstract

The stay-green (SG) trait enhances photosynthetic activity during the late grain-filling period, benefiting grain yield under drought and heat stresses. CH7034 is a wheat breeding line with SG. To clarify the SG loci carried by CH7034 and obtain linked molecular markers, in this study, a recombinant inbred line (RIL) population derived from the cross between CH7034 and non-SG SY95-71 was genotyped using the Wheat17K single-nucleotide polymorphism (SNP) array, and a high-density genetic map covering 21 chromosomes and consisting of 2159 SNP markers was constructed. Then, the chlorophyll content of flag leaf from each RIL was estimated for mapping, and one QTL for SG on chromosome 7D was identified, temporarily named *QSg.sxau-7D*, with the maximum phenotypic variance explained of 8.81~11.46%. A PCR-based diagnostic marker *7D-16* for *QSg.sxau-7D* was developed, and the CH7034 allele of *7D-16* corresponded to the higher flag leaf chlorophyll content, while the *7D-16* SY95-71 allele corresponded to the lower value, which confirmed the genetic effect on SG of *QSg.sxau-7D*. *QSg.sxau-7D* located in the 526.4~556.2 Mbp interval is different from all the known SG loci on chromosome 7D, and 69 high-confidence annotated genes within the interval expressed throughout the entire period of flag leaf senescence. Moreover, results of an association analysis based on the diagnostic marker showed that there is a positive correlation between *QSg.sxau-7D* and thousand-grain weight. Our results revealed a novel QTL *QSg.sxau-7D* whose CH7034 allele had a strong effect on SG, which can be applied in further wheat molecular breeding.

## 1. Introduction

The world population will reach approximately 10 billion by 2050. At the appointed time, the food demand is estimated to be 35~56% higher than that in 2010, and this number may increase to over 60%, considering the impact of extreme weather such as drought and high temperature due to global warming, which is needed for the significant improvement of grain production [1,2]. As one of the most widely grown crops in the world, wheat (*Triticum aestivum* L., 2n = 6x = 42) contributes about 20% of the total calories for the human diet and plays a key role in global food security [3]. Enhancements of the resistance to drought and heat of wheat varieties to cope with climate change and thereby increasing yield is an important goal for breeders worldwide, which requires a better understanding of the complex genetic architecture of grain yield and its constituent traits [3,4,5].

Stay-green (SG) trait, characterized by a longer green state of crop leaves in the late period of grain-filling, can provide more photosynthetic products, thereby increasing grain yield [6]. SG phenotype was initially described in *Vicia faba* breeding [7], and later on, it was established as an excellent feature for many grain crops such as sorghum [8], maize [9], rice [10], and barley [11]. In wheat, SG is associated with desirable morphological traits, including a higher number of tillers and grains per spike, enhanced resistance to stem lodging, and increased fertility of spikes [12,13,14]. Moreover, the beneficial roles of SG in wheat yield improvement under drought and heat stresses have been widely reported [15,16,17,18].

Physiologically speaking, SG is a manifestation of impaired or delayed chlorophyll catabolism in leaves. Research on chlorophyll synthesis and degradation pathways deepened our understanding of SG [19,20]. In plant leaves, chlorophyll *a* and chlorophyll *b* are synthesized through the magnesium (Mg) branch of tetrapyrrole biosynthesis and participate in photosynthesis. In this pathway, Mg^2+^ is inserted into the chlorophyll precursor, protoporphyrin IX, under the catalysis of magnesium chelating enzyme (MgCh), initiating the first step of biosynthesis. MgCh is composed of subunits CHLOROPHYLL I (CHLI), CHLD, and CHLH [21]. In wheat, the mutation of *TaCHLI* gene caused yellow-green leaves due to a decrease in chlorophyll content [22,23]. When the leaves age normally, the reactive oxygen species (ROS) increase and chlorophyll degrades in mesophyll cells, leading to leaf chlorosis. Furthermore, the chlorophyll degradation pathway involves chlorophyllase (CHLase)-catalyzed dephytylation of chlorophyll molecules into specific chlorophyllide followed by removal of the Mg^2+^ by Mg-dechelatase [24]. The expression level of wheat *TaCHLase* gene in the leaves of SG varieties was significantly lower than that of non-SG varieties [25]. Therefore, gene mutations in the above metabolic pathways, which cause a decrease in chlorophyll degradation or a sustained biosynthesis of chlorophyll in excess of the activity of the catabolic pathway, will result in the SG phenotype. The broad definition of SG includes five types [6]. Type-A and type-B belong to functional SG, while the other three types are considered cosmetic SG that are not interrelated with plant yield. Only type-A delays the onset of leaf senescence and prolongs the time of photosynthesis. For type-B, senescence occurs on schedule but proceeds relatively slowly. For the cosmetic SG, the senescence is initiated in the normal period; however, leaf greenness is maintained on the surface due to the failure of the chlorophyll degradation with weakened photosynthesis (type-C), or chlorophyll remaining because of freezing or drying (type-D), or the intensely green germplasm containing the high content of chlorophyll (type-E). Among the above-mentioned types, type-A can significantly increase photosynthetic products. In addition, due to the delayed senescence of type-A germplasm, the function of ROS scavenging in plant cells still exists. Therefore, compared with other SG types or non-SG germplasms, type-A germplasm shows better tolerance to abiotic stress such as high temperature and drought, which is the most important for agricultural production [26,27].

In the past decades, wheat breeders used forward genetics methods to screen SG with heritability and its linked molecular markers, in order to apply them to breeding. Multitudinous loci regulating SG were detected on all 21 chromosomes of wheat [28,29]. However, in some research, due to the adoption of relatively simple indicators such as grading scores for leaf color and duration of green leaves to record the SG phenotype [30,31], or the use of simple sequence repeats (SSR) markers for mapping [32,33,34], most SG loci showed low phenotypic variance explained (PVE) values or with distant linkage markers, which limits their breeding applications. With the development of biotechnology, chlorophyll meters have gradually been used in recent years to accurately measure the SG phenotypes of populations, and single nucleotide polymorphism (SNP) chips have also been used to perform genetic mapping. Currently, flag leaf chlorophyll content is used as an important phenotype data for quantitative trait locus (QTL) mapping for SG based on a high-density genetic map. In a recombinant inbred line (RIL) population containing 309 lines derived from the cross of spring wheat cultivars Worrakatta and Berkut, one QTL for chlorophyll content, *QCC.xjau-1DS*, was identified on chromosome 1D with the phenotypic variation explained (PVE) ranging from 5.3% to 5.8% [35]. In another RIL population consisting of 165 lines derived from winter wheat cross DH118 × Jinmai 919, four QTLs for chlorophyll content, including *Qchl.saw-3B.2*, *Qchl.saw-5A.2*, *Qchl.saw-5A.3*, and *Qchl.saw-3B.2*, were detected, and the PVE ranged from 5.91% to 7.24% [36]. Moreover, in a doubled haploid (DH) population with 201 lines constructed by the winter wheat crossing Jinmai 47 × Jinmai 84, six major QTLs *Qchl.saw-2B.1*, *Qchl.saw-3B.1*, *Qchl.saw-4D.1*, *Qchl.saw-4D.2*, *Qchl.saw-5A.9*, and *Qchl.saw-6A.4* were detected under multiple phosphorus conditions, with the PVE ranging from 3.07% to 31.66% [37]. In winter wheat Hanxuan10 × Lumai14 DH population containing 150 lines, 28 QTLs for chlorophyll content with the PVE ranging from 5.8% to 21.4% were identified on 11 chromosomes [38].

Here, we reported an SG wheat breeding line CH7034. A RIL population was created via the cross of CH7034 and a non-SG breeding line, and QTL for chlorophyll content of flag leaf was detected based on a high-density genetic map, in order to provide germplasm resources tolerant to stress and related molecular markers for wheat breeding.

## 2. Material and Methods

### 2.1. Plant Materials and Experimental Conditions

The SG wheat breeding line CH7034 (CH) was bred by the College of Agronomy of Shanxi Agricultural University, and the non-SG breeding line SY95-71 (SY) was bred by the Triticeae Research Institute of Sichuan Agricultural University. CH and SY were crossed to generate an RIL population by single seed descent from the F_2_ individual plants [39], and a set of 184 RILs was planted in the greenhouse at Shanxi Agricultural University (Jinzhong, China) in 2019, 2023, and 2024. In early November of the three years mentioned above, fifteen seeds from each RIL and the parents were planted in a row of 1.5 m in length, with a plant spacing of approximately 10 cm.

### 2.2. Phenotyping of SG

The chlorophyll content of the flag leaf was measured to evaluate SG capability. In brief, ten plants were randomly selected from each RIL and the parents at the grain-filling stage, and the relative chlorophyll content, represented as the soil and plant analyzer development (SPAD) value, was measured in the middle of the flag leaves by using chlorophyll meter SPAD-502 Plus (Konica Minolta Sensing Inc., Tokyo, Japan). An average of SPAD value on 10 plants was numerated and recorded for each RIL and the parents, and the best linear unbiased estimation (BLUE) values of SPAD data in three years were finally calculated by Genstat (version 22) software.

### 2.3. Genotyping and QTL Mapping

Genomic DNA was extracted from leaves of RILs and their parents at seedling stage and then genotyped by a Wheat17K SNP chip containing 17,526 markers (Diversity Arrays Technology Pty Ltd., Canberra, Australia). For the genotypic data, the parent-polymorphic SNP markers with less than 20% missing values and precise positioning information on the Chinese Spring genome (version 1.0, International Wheat Genome Consortium, Eau Claire, WI, USA) were screened to construct linkage groups by running the MAP program of IciMapping (version 4.0) software. Then, integrating with SPAD–BLUE values of RILs, QTL analysis was performed using the Kosambi function under the BIP program of ICIMapping software, setting the threshold value of the logarithm of the odds as 2.5.

### 2.4. Diagnostic Marker Developing

A PCR-based diagnostic marker for SG was developed as described previously [40]. First, SSR loci within the target genome segment were searched by SSRHunter (version 1.3) software, and specific primers were designed for these loci (Table 1). Afterward, primer pairs were used for amplifying the genomic DNA of CH and SY, and then the markers with parental polymorphism were screened for genotyping in the RIL population. The amplification condition was as follows: 94 °C for 30 s, 36 cycles of 94 °C for 30 s, 58 °C for 30 s, and then 72 °C for 30 s, with a final extension of 72 °C for 3 min. Polyacrylamide gel electrophoresis was used for differentiating the PCR products. Finally, these SSR markers were integrated into the QTL map, and the marker under the peak was used as the diagnostic marker.

### 2.5. Expression Pattern of Annotated Genes Within QTL

A set of publicly available transcriptome data [41] involving wheat flag leaves at different days after anthesis (DAA) was used for analyzing the expression patterns of high-confidence annotated genes within target QTL, as described previously [42]. In brief, the transcriptome data were downloaded from the National Center for Biotechnology Information (NCBI) database (www.ncbi.nlm.nih.gov, accessed on 31 December 2024) using accession number PRJNA497810. Filtered reads were mapped to the Chinese Spring genome (version 1.0), and the number of transcripts aligned to each gene was calculated and normalized to transcripts per kilobase million (TPM) values. Then, the TPM values of annotated genes were selected to generate a heatmap on the SRplot platform (www.bioinformatics.com.cn/SRplot, accessed on 10 January 2025).

### 2.6. Association Analysis Between SG-QTL and Agronomic Traits

A diagnostic marker of SG-QTL was used to conduct differential analysis for agronomic phenotypes corresponding to the two SG-QTL alleles, as described previously [43]. Phenotypic data were obtained from the CH × SY RIL population in 2024, which was planted in the greenhouse at Shanxi Agricultural University as described in Section 2.1. For spike traits, the spike number per plant, spike length, and spikelet number per spike were investigated. For grain traits, the thousand-grain weight, grain length, grain width, and grain area were evaluated using the TPKZ-3 intelligent seed test and analysis system (Top Cloud-Agri Technology Co., Ltd., Hangzhou, China).

### 2.7. Statistical Analysis

The Origin (version 3.1) software was used to perform the statistical analysis by one-way analysis of variance (ANOVA), and *p* < 0.05 was considered a significant difference, while *p* < 0.01 was considered an extremely significant difference.

## 3. Results

### 3.1. Assessment of SG for CH and SY

The two breeding lines CH and SY have large differences in leaf color at the grain-filling stage. CH showed the SG phenotype and the chlorophyll content of its flag leaves ranged from 3.43 to 4.12, whereas SY showed aging and yellow flag leaves with a chlorophyll content of 42.27~50.39. The BLUE value of chlorophyll content in CH was significantly higher than those in SY (*p* < 0.0001) (Table 2).

### 3.2. Phenotypic Variance of SG in the CH × SY RILs

**Figure 1:**
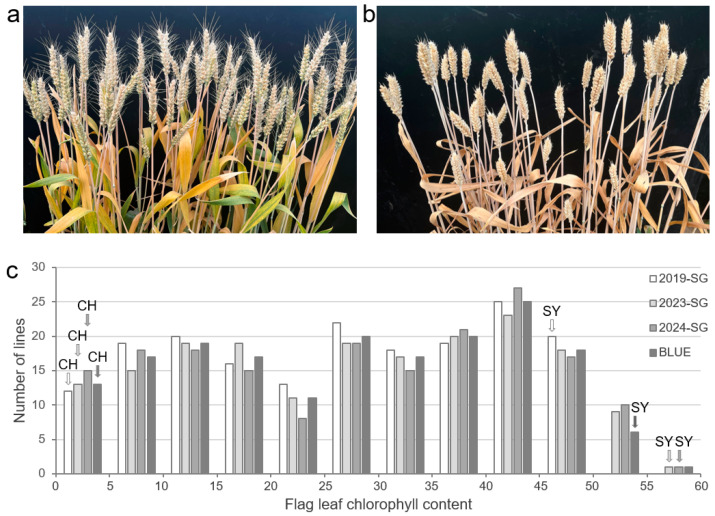
Variation in stay green in CH × SY RIL population. (**a**) Stay-green line. (**b**) Non-stay-green line. (**c**) Flag leaf chlorophyll content of RILs. CH: CH7034; SY: SY95-71; SG: stay green; BLUE: best linear unbiased estimation.

SG showed segregation in the RIL population derived from the cross of CH and SY, which ranged from 1.66 to 57.28, with the coefficient of variation values of 0.32~0.56 (Figure 1, Table 2). According to the phenotypic data of three years and the derived BLUE dataset, flag leaf chlorophyll content in the RILs exhibited continuous distribution (not normal distribution) and transgressive segregation, indicating that SG was a complex trait controlled by multi-genes (Figure 1).

### 3.3. Genetic Mapping of SG

**Figure 2:**
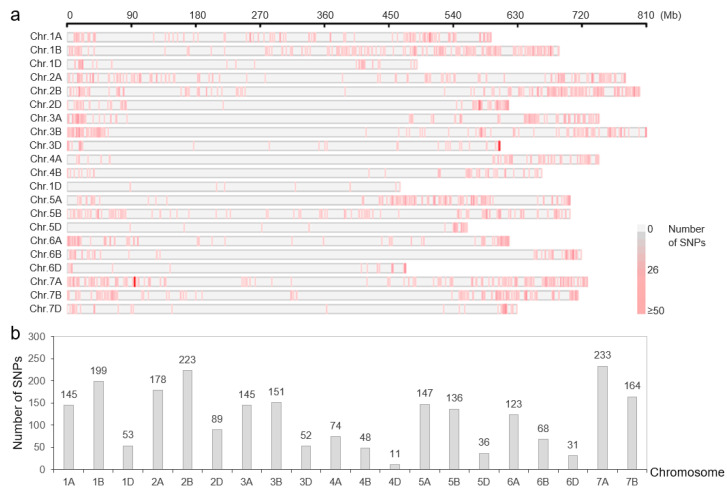
SNPs from the 17K SNP chip screened to form a genetic map for the RILs. (**a**) Distribution of SNPs on wheat chromosomes. (**b**) The number of SNPs on each chromosome.

A total of 2359 SNPs with parental polymorphism from the 17K SNP chip were screened after removing the redundant or excessive missing data (Figure 2). These SNP markers were assembled into 21 chromosomes to form a genetic map for the RILs with a marker density of 1.94 cM per marker. Based on the linkage map and the phenotypic data, two genomic regions on chromosomes 5A and 7D were found to have significant effects on the SG trait in 2019, with a PVE of 5.77% and 11.46%, respectively; two genomic regions on chromosomes 2B and 7D involving SG were identified in 2023, with a PVE of 5.24% and 10.62%, respectively; and one genomic region on chromosome 7D with a PVE of 8.81% was found to have significant effects on the SG trait in 2024 (Table 3). Among them, the QTL on chromosome 7D, temporarily named *QSg.sxau-7D*, was detected in the three years and with the PVE of 10.33% based on the BLUE value (Table 3).

### 3.4. Verification of QSg.sxau-7D

**Figure 3:**
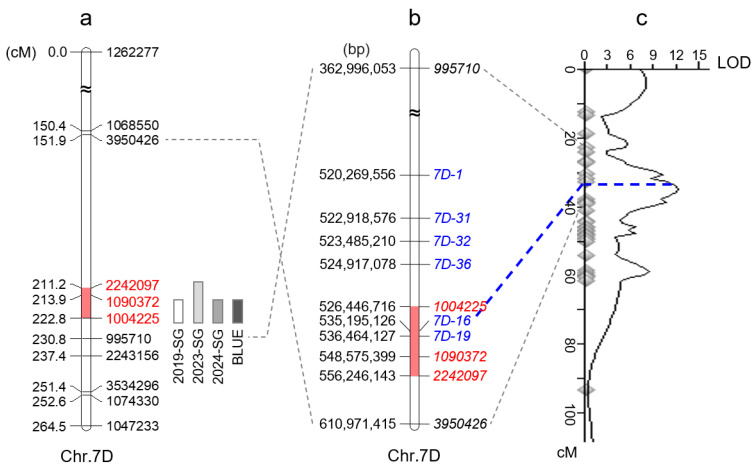
Map position and linked markers of *QSg.sxau-7D*. (**a**) Genetic map. (**b**) Genomic map. (**c**) Re-mapping result of *QSg.sxau-7D* integrated with six developed SSR markers. Red boxes represent *QSg.sxau-7D* region; the linked SNP markers are marked in red, while the developed SSR markers are marked in blue.

To verify the mapping results, PCR-based markers for *QSg.sxau-7D* were developed. *QSg.sxau-7D* was mapped to a 11.6 cM interval flanked by markers *1004225* (chr.7D: 526,446,716) and *2242097* (chr.7D: 556,246,143), corresponding to a physical range of 29.8 Mbp (Figure 3a,b). We randomly designed SSR primer pairs within the genome segment that ranged from 10 Mbp upstream of *1004225* to 10 Mbp downstream of *2242097*, and six pairs of primers, including *7D-1*, *7D-31*, *7D-32*, *7D-36*, *7D-16*, and *7D-19*, were showed polymorphisms between CH and SY. These parent-polymorphic markers were used for amplifying the CH × SY RIL population and finally integrating into *QSg.sxau-7D* map. The SSR marker *7D-16* under the peak of *QSg.sxau-7D* was used as the diagnostic marker (Figure 3c).

### 3.5. Annotated Genes Within QSg.sxau-7D

**Figure 4:**
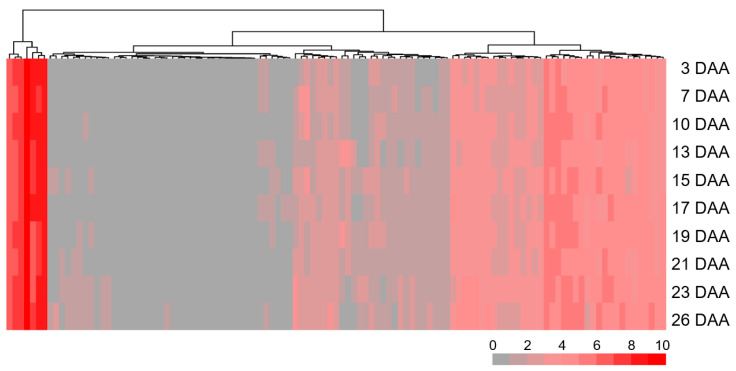
Expression patterns of 280 annotated genes within *QSg.sxau-7D* region in wheat flag leaf from the main tiller that harvested at 3, 7, 10, 13, 15, 17, 19, 21, 23, and 26 days after anthesis (DAA). Unexpressed genes are represented by gray squares. From gray to red, the TPM value goes from low to high.

There are 280 high-confidence genes in the *QSg.sxau-7D* interval (chr.7D: 526,446,716–556,246,143) according to the annotation of coding sequences (RefSeq v1.1) in the Chinese Spring genome database (Table S1). Based on published data [41], 167 annotated genes were not expressed in wheat flag leaf from the main tiller after anthesis. The remaining 113 expressed genes mainly involved DNA binding or protein binding in the category of molecular function based on their gene ontology annotation (Table S1), and 69 of which were expressed throughout the entire period of flag leaf senescence, including *TraesCS7D01G409100* encoded Myb transcription factor (MYB), *TraesCS7D01G415100* encoded zinc finger CCHC-type protein (ZCCHC), and *TraesCS7D01G409700* and *TraesCS7D01G436800* encoded auxin response factor (ARF) (Figure 4, Table S2).

### 3.6. The Correlation Between QSg.sxau-7D and Agronomic Traits

**Figure 5:**
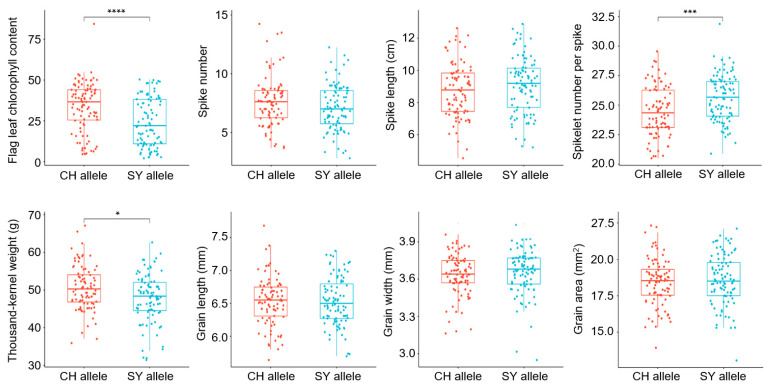
Significant differences analysis in genotypes of the diagnostic marker *7D-16* of *QSg.sxau-7D* in CH × SY RIL population. * indicates *p* < 0.05, *** indicates *p* < 0.001, and **** indicates *p* < 0.0001, by the *t*-test.

Based on the phenotypic data of the CH × SY RIL population in 2024, the CH allele of the diagnostic marker *7D-16* corresponded to the higher flag leaf chlorophyll content, while the *7D-16* SY allele corresponded to the lower value (*p* = 2.09 × 10^−5^), which confirmed the effect on SG of *QSg.sxau-7D* (Figure 5). Furthermore, *7D-16* was also used for testing the effects of *QSg.sxau-7D* on agronomic traits. For spike traits, RILs carrying *QSg.sxau-7D* CH allele showed a lower spikelet number per spike than that carrying the SY allele (*p* = 0.00013). There are no significant correlations between *QSg.sxau-7D* and spike number per plant (*p* = 0.09) or spike length (*p* = 0.22). For grain traits, RILs carrying the *QSg.sxau-7D* CH allele showed a higher thousand-grain weight than that carrying the SY allele (*p* = 0.03). No significant correlation was shown between *QSg.sxau-7D* and grain length (*p* = 0.97), grain width (*p* = 0.24), as well as grain area (*p* = 0.54).

## 4. Discussion

### 4.1. QSg.sxau-7D Is a Novel SG Locus

**Figure 6:**
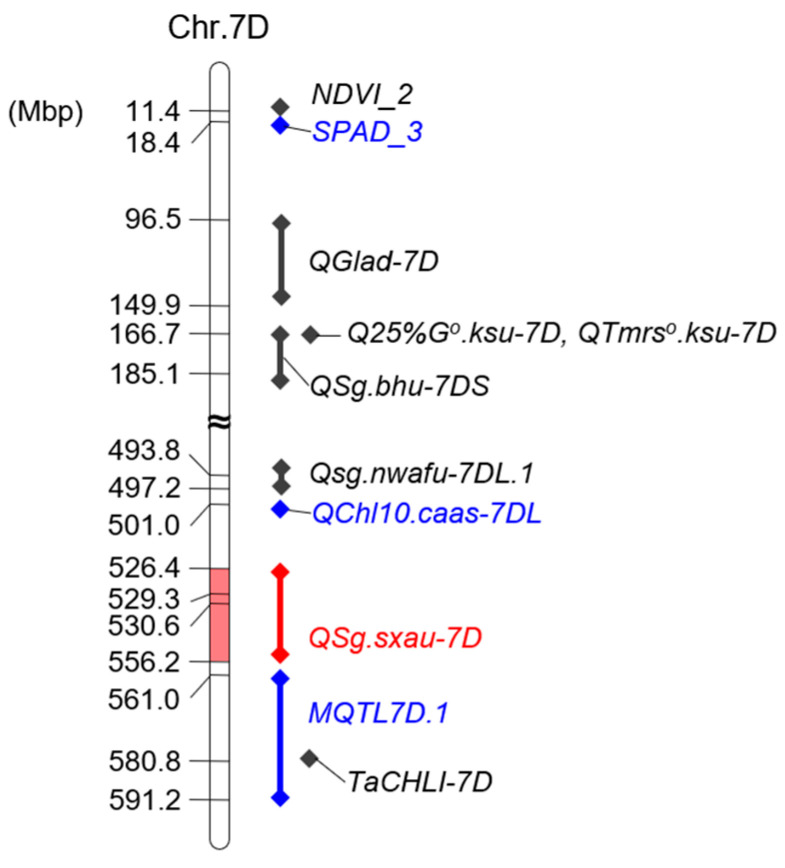
The physical positions of *QSg.sxau-7D* and the previously reported SG-associated loci on chromosome 7D. The line with rhombic dots indicates QTL interval flanked by markers, and the single rhombic dot indicates the solitary linked marker of QTL. QTLs for flag leaf chlorophyll content that were measured by SPAD value are marked in blue, and the *QSg.sxau-7D* in this study is marked in red.

*QSg.sxau-7D* was located in the chr.7D:526.4~556.2 Mbp physical position on the long arm of chromosome 7D. Currently, a total of nine loci related to SG, which were evaluated by SPAD value, normalized difference vegetation index (NDVI), green leaf area duration (GLAD), or leaf area under greenness (LAUG), have been reported on chromosome 7D (Figure 6). Among them, *NDVI_2*, *SPAD_3* [44], *Qglad-7D* [45], *Q25%G^o^.ksu-7D*, *Qtmrs^o^.ksu-7D* [46], and *QSg.bhu-7DS* [47] were mapped on the short arm of chromosome 7D. Moreover, on the chromosome long arm, *Qsg.nwafu-7DL.1* was located in the chr.7D:493.8~497.2 Mbp interval [48], *QChl10.caas-7DL* was located in the chr.7D:501.0 Mbp position [49], while *MQTL7D.1* was mapped to the physical locations of the chr.6B:561.0~591.2 Mbp [29] that contained a chlorophyll synthesis gene *TaCHLI-7D* [23]. In summary, the locations of all reported SG-QTLs mentioned above are different from those of *QSg.sxau-7D*, so *QSg.sxau-7D* should be a novel SG-locus. In addition, a QTL for drought survival rate (DSR) [50] was located in the *QSg.sxau-7D* region. It was reported that SG enhances the drought tolerance of wheat [15,16,18], so, we will further investigate whether *QSg.sxau-7D* is associated with drought resistance.

### 4.2. Prediction of Candidate Genes for QSg.sxau-7D

There are 280 high-confidence genes in the *QSg.sxau-7D* interval according to the annotation of coding sequences in the Chinese Spring genome, 69 of which were expressed in wheat flag leaf throughout the entire period of senescence, including several genes encoding transcription factors such as *TaARFs* (*TraesCS7D01G409700* and *TraesCS7D01G436800*), *TaZCCHC* (*TraesCS7D01G415100*), and *TaMYB* (*TraesCS7D01G409100*).

It has been established that ARF plays an important role in the regulation of SG in wheat [51,52]. An ARF member *TaARF15-A1* delays leaf senescence by positively modulating senescence-delaying genes such as the NAC gene *TaNAM-1*. *TaARF15-A1*-overexpression plants showed the SG phenotype [52]. According to the wheat ARF family number [53], the two ARF genes in this study were named as *TaARF20-D* and *TaARF21-D*, and we will conduct in-depth research on them. Similarly, a CCCH-type Zinc finger gene, *Strong Staygreen* (*SSG*), was characterized as the suppressor of leaf senescence as overexpression or suppression of the gene led to delayed or accelerated leaf senescence, respectively, in switchgrass (*Panicum virgatum* L.) [54]. In addition, MYB proteins have been reported to be involved in promoting chlorophyll catabolism. The MYB-related transcription factor RADIALIS-LIKE3 (OsRL3) functions in ABA-induced leaf senescence in rice, and the *osrl3* null mutants exhibited a stay-green phenotype in the dark, with increased chlorophyll retention and photosynthetic capacity [55]. Proteasomal degradation of MaMYB60 caused high temperature-induced repression of chlorophyll catabolism and stay-green peels in bananas [56].

### 4.3. CH7034 Can Be Used for Wheat Breeding

SG trait of wheat can provide more photosynthetic products in the late period of grain-filling. In this study, we reported an SG wheat breeding line CH7034 and detected *QSg.sxau-7D* for flag leaf chlorophyll content with the BLUE-PVE of 10.33%. Association analysis results showed that the SG-type *QSg.sxau-7D* CH allele corresponded to the increased thousand-grain weight, comparing the non-SG-type SY allele, displaying a good application prospect in wheat high-yield breeding. However, RILs carrying the *QSg.sxau-7D* CH allele showed decreased spikelet number per spike than that carrying the SY allele. This is because a QTL for spike number per spike, *QSns.sxau7D* [57], is near *QSg.sxau-7D*. *QSns.sxau-7D* explained up to 17.92% of the total phenotypic variation in five experimental locations/years, and the spikelet number per spike of the *QSns.sxau-7D* CH allele was lower than that of the SY allele [57]. In addition, CH7034 is an excellent germplasm resistant to abiotic stress such as drought and salt [58]. A QTL *DSR* involving drought tolerance [50] was located in the *QSg.sxau-7D* region, suggesting the possible effects on the abiotic stress response. By marker development, the PCR-based *7D-16* under the peak of *QSg.sxau-7D* was used as the diagnostic marker, which can be applied in wheat molecular breeding.

## 5. Conclusions

This study clarified the SG loci carried by CH7034 and obtained close-linked molecular markers. One QTL for flag leaf chlorophyll content, *QSg.sxau-7D*, was detected in the genomic interval 526.4~556.2 Mbp on chromosome 7D, with the maximum PVE of 11.46%. There were 280 high-confidence genes in the interval. *QSg.sxau-7D* was positively correlated with thousand-grain weight. One PCR-based diagnostic marker *7D-16* for *QSg.sxau-7D* was developed for application in wheat molecular breeding.

## Figures and Tables

**Table 1:** Developed SSR markers linked to QTL in this study.

Marker	Position	Forward Primer Sequence (5′–3′)	Reverse Primer Sequence (5′–3′)
*7D-1*	7D:520269556	ATAGATTTGACTTTGGGGTAGTAG	TCTCAGAGAGCAACTTGGG
*7D-31*	7D:522918576	TAGCTCCCTCCAGTGAAAC	CCTGCTCCAAACCCAATCT
*7D-32*	7D:523485210	ATGCTTCCACGCCGGTGT	AGACATCAACCTTTCCCAGATC
*7D-36*	7D:524917078	ACTGCTCTCTCTATTATAGCAG	GCTTTGACATGGTCTAAATCTG
*7D-16*	7D:535195126	GGACTCGGACGCCAAAGT	CCAGTTTCGAGTCTCGTTTTTTA
*7D-19*	7D:536464127	CAAAACATGGGCTAGATCCC	CTAGATCTACAGGCCAACAC

**Table 2:** Assessment of stay green for CH × SY RIL population and the parents.

Trait	Parents		RIL Population			
	CH	SY	Min	Max	Mean	CV
2019-SG	44.17	4.12 ****	2.33	49.43	29.11	0.32
2023-SG	50.39	3.84 ****	2.69	55.74	30.04	0.47
2024-SG	42.27	3.43 ****	1.66	57.28	28.01	0.56
BLUE	45.61	3.80 ****	3.05	52.86	31.37	0.44

SG: stay green; CV: coefficient of variation; BLUE: best linear unbiased estimation; ****: *p* < 0.0001 by the *t*-test.

**Table 3:** QTL mapping for stay green in CH × SY RIL population.

Trait	Chromosome	Position (cM)	Left Marker	Right Marker	LOD	PVE (%)	ADD
2019-SG	5A	198.5	2260698	1234116	2.84	5.77	5.39
	7D	213.9	1004225	1090372	5.27	11.46	12.54
2023-SG	2B	170.2	5411126	1863181	2.63	5.24	2.47
	7D	211.2	1004225	2242097	5.06	10.62	12.24
2024-SG	7D	213.9	1004225	1090372	3.50	8.81	4.81
BLUE	7D	213.9	1004225	1090372	5.57	10.33	9.26

SG: stay green; LOD: logarithm of the odds; PVE: phenotypic variance explained; ADD: additive effect.

## Data Availability

Data are contained within this article and Supplementary Materials.

## Supplementary Materials

The supporting information can be downloaded at: https://www.mdpi.com/article/10.3390/plants14050727/s1, Table S1: 280 high-confidence annotated genes within *QSg.sxau-7D* region; Table S2: Expression patterns of 280 annotated genes in wheat flag leaf harvested at 3, 7, 10, 13, 15, 17, 19, 21, 23, and 26 days after anthesis (DAA).
